# Drugs and gut microbiome interactions—an emerging field of tailored medicine

**DOI:** 10.1186/s40360-023-00684-9

**Published:** 2023-08-30

**Authors:** Imran Khan

**Affiliations:** Department of Biotechnology, Faculty of Chemical and Life Sciences, Abdul Wali Khan University Mardan, Mardan city, 23200 Pakistan

## Abstract

Gut dwelling microbes provide profound biochemical advantages to the host, including nutrient and drug absorption, metabolism, and excretion. It is an emerging understanding that drug-response bias (particularly for orally intake medicine) is related to variation in the microbial composition in the gut. This Editorial at BMC Pharmacology and Toxicology introduces our collection which is discussing the role of gut microbes in modulating drugs’ efficacy and bioavailability.

## Introduction

Humans have co-evolved with microbes that constitute about 43% of the human body by cell count. Any dysbiosis in the microbial composition could put the host at risks of developing diseases like obesity, inflammatory bowel disease, diabetes, rheumatoid arthritis, and cancer [1–4]. These microbes inhabit the exterior and interior surfaces of the body. Among the body parts, gastrointestinal tract (GIT) is the most densely populated by microbes, known as gut microbiota (GM), which provides significant biochemical advantages to the host in nutrient and drug absorption, metabolism, and excretion.

GM influences a wide range of body’s function. Variation in the GM diversity and composition result in a profound impact on host’s metabolism, immunity, and response to orally consumed drug. It is an established fact that intra-individual variability in the composition of GM contributes to drug response biases for certain therapeutics. One might have noticed why some drugs are more efficient in some individuals than others. It has been speculated that these drug biases might be due to the variations in the GM diversity and composition of the patient. As drugs pass through the GIT and come into contact with numerous microbial enzymes, they undergo extensive structural modification that consequently affect their function. There are several mechanisms through which GM could remodel drugs’ efficacy and bioavailability.

### GM improve drugs’ efficacy through immune system

One such example is the anti-PD-L1 antibodies (a treatment for cancer) treatment, which is dependent upon the composition of gut microbiota. It is observed that anti-PD-L1 (an antibody that blocks CTLA-4) efficacy improves in the presence of *Bifidobacterium*. Oral administration of anti-PD-1 and *Bifidobacterium* has been observed with augmented dendritic cell function and improved CD8^+^ T cell priming in the tumor microenvironment [5]. Besides, anti-CTLA-4 therapies have also shown dependence on the presence of *Bacteroides thetaiotaomicron* or *Bacteroides fragilis*. In another study, it was noticed that melanoma patients harboring more *Ruminococcaceae* responded effectively toward anti-CTLA-4 treatments [6].

Another such an example is cyclophosphamide, an anti-cancer immune-suppressant chemo-therapeutic, which efficacy is dependent on *Barnesiella intestinihominis* and *Enterococcus hirae* (gut commensals) that contribute to the translocation of lymphoid organ and mimic the production of pathogenic T helper 17 cells and memory Th1 immune responses [7]. During cyclophosphamide therapy, *Barnesiella intestinihominis* accumulates in the colon and infiltrates γδT cells in the cancer lesion. Whereas *Enterococcus hirae* translocates to secondary lymphoid organs and stimulates intratumoral CD8/Treg ratio [8]. Microbial influences on the efficacy of chemotherapeutic drugs, 5-fluoro-2′-deoxyuridine, and 5-fluorouracil have also been reported [9].

### GM affect drug’s efficacy through biotransformation

In addition to the mechanisms discussed above, GM could also remodel drug’s efficacy through biotransformation [10]. For instance, *Egertella* species (gut-dwelling bacteria) reduce digoxin (a cardenolide for treating heart failure) to an active metabolite (called dihydrodioxin) through an enzyme called Cgr2 [11]. Similarly, levamisole (a drug use for treating colon cancer) is transformed into levametabol-I, II and III by gut bacteria [12]. Therefore, it is important to evaluate the function of GM-generated metabolites. In some cases, GM encoded enzymes could remodel the structure of a drug in a way that causes severe side effects. For example, CPT-11 is colon cancer chemotherapeutic which is transformed by the β-glucuronidases of the gut commensal and can cause severe diarrhea. To tackle these unwanted outcomes, designing targeted inhibitors with the aim of blocking the enzymes rather than eradicating gut commensals is essential. Study looking into this has previously been done by Wallace et al. where they developed bacterial β-glucuronidase inhibitors that had no effect on the orthologous mammalian enzyme [13].

### GM affect drug’s availability through bioaccumulation

In process of bioaccumulation, bacteria accumulate drug intracellularly without changing its structure or growth. Thus, restricting the drug availability to the host. For instance, the bioavailability of antidepressant drug duloxetine and the antidiabetic drug rosiglitazone is significantly affected by *Enterococcus* sp., *Lactobacillus* sp., and *Bifidobacterium* sp. through the process of bioaccumulation [10]. Another study has reported the bioaccumulation of simvastatin in an in vitro setting [14].

In conclusion, GM is emerging as a new player in personalized medicine, and various methods are being developed to treat diseases by remodeling of patients’ GM composition. However, it is emphasized that microbiome-based therapies should be tailored to disease types and body sites. For example, men with metastatic prostate tumors who responded to checkpoint inhibition have been found to have lower levels of *Akkermansia muciniphila* in their stool than men who did not respond. But the opposite is true for people with lung and kidney cancers — those with more *A. muciniphila* in their guts tended to fare better on the therapy [15]. This collection on Drugs and gut microbiome interactions, launched in BMC Pharmacology and Toxicology aims to highlight new interaction of drug-GM, and how these interactions can improve drug efficacy and bioavailability. We also welcome studies that investigate gut bacteria that that are responsible for in activation of drugs.

## Data Availability

Not applicable.

## List of abbreviations

GM: Gut microbiota
GIT: Gastrointestinal tract
